# The Correlation between the Frontostriatal Network and Impulsivity in Internet Gaming Disorder

**DOI:** 10.1038/s41598-018-37702-4

**Published:** 2019-02-04

**Authors:** Jin-Young Kim, Ji-Won Chun, Chang-Hyun Park, Hyun Cho, Jihye Choi, Siyun Yang, Kook-Jin Ahn, Dai Jin Kim

**Affiliations:** 1 Department of Psychiatry, Seoul St. Mary’s Hospital, The Catholic University of Korea College of Medicine, Seoul, Republic of Korea; 2 Department of Psychology, Korea University, Seoul, Republic of Korea; 3 Department of Radiology, Seoul St. Mary’s Hospital, The Catholic University of Korea College of Medicine, Seoul, Republic of Korea

## Abstract

As excessive use of internet gaming has become a serious public health concern, increasing studies have revealed that impulsivity is one of the important risk factors of internet gaming disorder (IGD). This study was designed to investigate the altered resting-state functional connectivity (FC) of the bilateral orbitofrontal cortex (OFC) in IGD participants and to examine its relationship with impulsivity compared with the normal controls (NC). Seed-based analyses verified that participants with IGD displayed decreased FC between the OFC and frontal, striatal, temporal and occipital regions different from NC. Moreover, IGD participants showed weankened FC from the OFC with dorsal anterior cingulate cortex as well as with dorsolateral prefrontal cortex and dorsal striatum as the results of group difference. These results could suggest that the decreased frontostriatal connectivity was associated with excessive internet gaming. Also, the increased FC in frontostriatal regions was correlated with impulse control in the NC but not the IGD participants. Further insight into the brain circuitry on frontostriatal could provide the target for developing treatment approaches of impulse control in IGD.

## Introduction

The internet game market has grown fast so that the breadth of the market was nearly 100 billion dollars in 2016 with the development of internet service and portable devices. Some Asian countries, including South Korea, have a higher prevalence of internet gaming disorder than is found in North American or European countries, perhaps because of the rapid development of communication networks^1,2^. There is no question that the world has gone through problems with internet game usage as more personal and social problems have been turning up^3^. However, it is unclear whether internet gaming disorder (IGD) is its own addictive disorder or only a psychotic symptom of another psychosis as its behavioral and psychiatric symptoms are similar to those of the other addictive disorders^4^. With a need to clarify its symptoms, IGD was included in the Diagnostic and Statistical Manual of Mental Disorders, Fifth Edition (DSM-5) section 3 as a tentative disorder that needs further research^5^. Nine IGD criteria were suggested in DSM-5 on the basis of the gambling disorder and substance use disorder criteria for preliminary studies. The criteria include typical clinical features of addictive disorder such as withdrawal, loss of control, and functional impairment^6^.

A few studies have indicated that playing online games exercises several cognitive functions^7^; however, most studies have reported disturbances in behavior, emotion and cognitive function as a consequence of excessive use of internet games. In particular, various studies shed light on impulsivity as an important trait of IGD. Impulsive behavior is the tendency of premature act without foresight^8^ which is considered as failure of cognitive control. Therefore, impulsivity predisposes individuals to lose their willpower for stopping addictive behavior, relapse^9^. IGD participants’ impulsive responses were seen during behavior task trials^10–12^. Additionally, a longitudinal study marked impulsivity as an important risk factor for addictive behavior for becoming a pathological gamer^13^.

According to recent functional magnetic resonance imaging (fMRI) studies, addiction is deeply associated with abnormal brain functional connectivity (FC) in cortico-striatal substrates, which leads people to be more impulsive^8,14–16^. Impaired FC of the frontostriatal circuitry^17,18^, which is comprised of prefrontal cortex (PFC), dorsal anterior cingulate cortex (dACC) and dorsal striatal regions, was associated with compulsive drug taking and impulsive behavior of addiction^19,20^. It was confirmed that the loss of control, a symptom of addictive disorders, was related to prefrontal/orbitofrontal-striatal circuitry in previous study^17^. IGD studies especially showed the association between frontal cortex among the frontostriatal regions and cognitive control which leads to impulsive control^21,22^. Neuro-circuitry changes in the prefrontal cortex, especially in the OFC, account for neural mechanisms of impaired decision-making and impulse control^23–28^, maladapted decision-making^29,30^ and deviant social behavior in compulsive gambling and drug addiction disorder^31–33^. Studies with fMRI also noted increased activity of the OFC when IGD participants were engaged in diverse tasks for decision-making and impulse control^21,34^. In addition to the frontal cortex, the dorsal striatum is also responsible for movement execution, decision-making and the inhibition of impulsivity^35,36^. The main results with the structural and functional frontostriatal connectivity from previous study are related to the impulse control, mainly with ADHD^37^. In studies, it was revealed that abnormal FC in the frontostriatal circuitry implies increased impatience and inattention and impulsivity^38,39^.

Despite abnormal FC within the frontostriatal networks being strongly related to self-control ability and impulsive behavior^40–42^, few studies have examined the relationship between the frontostriatal FC and impulsivity in IGD thus far. In particular, in spite of the well-known role of the OFC in behavior regulation and impulse control for addictive behavior^23,43–46^, it still remains unclear whether the FC between the OFC would affect the relationship with impulsivity in IGD. Lesion studies with human and also rodents revealed that deficit in the OFC showed more impulsiveness than normal control subjects and guided impaired goal-directed behavior and impulsive behavior^31,33,47^. Imaging studies indicated hypoactivity of the OFC during withdrawal in substance use addiction^48,49^. Thus we postulated that the OFC is responsible for impulse control over addictive behavior amongst frontostriatal network. As we have highlighted role of the OFC as impulse control, we made the OFC as a seed region so that we can find its functional relationship with the other brain regions and their engagement to impulsive level. Seed-based analysis is one way to assess connected coherent spontaneous fluctuation pattern of resting-state functional MRI signals between brain areas^50^. Based on the time series of the OFC, in this study, we calculated the correlated time series with other regions in the brain. We could find functionally connected area with the OFC with this analysis, so that the FC pattern would give us information that could compare between groups. We hypothesized that IGD participants would exhibit weakened FC from the bilateral OFC compared to the normal controls (NC), and we supposed that the altered FC, especially in the frontostriatal network components, would be related to impulsivity.

## Results

### Demographic and clinical data

The demographic and clinical characteristics are summarized in Table 1. The two groups did not differ in age, educational attainment or intelligence. The duration of the internet gaming was also not different between the groups, whereas IGD participants spent more money, *t*(44) = 2.70, *p* < 0.05, and time, *t*(44) = 5.72, *p* < 0.001, on internet game than NC. The IGD-scale score, *t*(44) = 13.53, *p* < 0.001, and impulsivity score, *t*(44) = 7.12, *p* < 0.001, were significantly higher in participants with IGD compared with NC.

**Table 1:** Demographic and clinical characteristics of participants.

	IGD (n = 22)	NC (n = 24)	t-value
**Demographic characteristic**			
Age	28.27 ± 5.33	28.17 ± 5.93	0.06
Years of education	15.09 ± 1.69	15.13 ± 1.62	−0.07
K-WAIS	108.36 ± 11.71	114.25 ± 9.73	−1.86
**Gaming characteristics**			
Years of internet game use	16.23 ± 3.07	15.00 ± 3.76	1.22
Hours of internet game use (/week)	26.41 ± 11.38	10.83 ± 6.04	5.72***
Cost for internet game (/month, KRW)	46955 ± 60966	10042 ± 20936	2.67*
**Clinical characteristic**			
Internet Gaming Disorder Scale	5.64 ± 1.84	0.21 ± 0.42	13.53***
Dickman’s Dysfunctional Impulsivity Inventory	6.5 ± 2.54	2.00 ± 1.69	7.12***

Abbreviations: IGD, Internet gaming disorder; NC, Normal control; K-WAIS, Korean version of Wechsler Adult Intelligence Scale.

*p < 0.05, **p < 0.005, ***p < 0.001.

### Functional MRI results

#### Regional differences between groups

**Figure 1:**
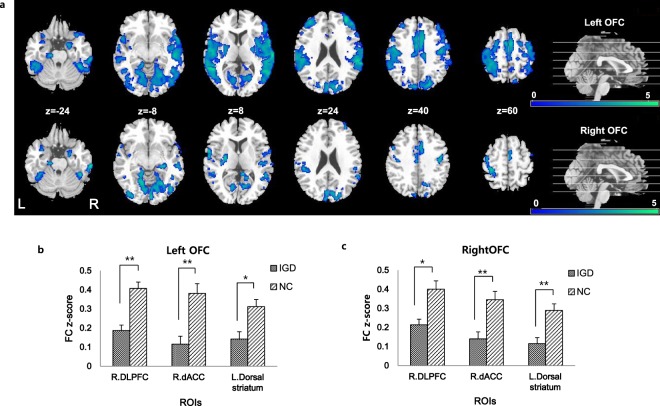
Participants with internet gaming disorder (IGD) showed (**a**) decreased functional connectivity (FC) from the bilateral seed region compared to the normal controls (NC) (*p*_*FDR*_ < 0.05, *k* > 100). FC from the left seed region (**b**) and right seed region (**c**) to each region of interest (ROI) was significantly low in IGD participants compared to the NC (*p < 0.05, **p < 0.001).

Seed-based FC maps comparing two groups are shown in Fig. 1 and Table 2 with a significance level of *p*_FDR_ < 0.05, k > 100. Generally, IGD participants showed weakened connectivity of the bilateral OFC with the other cerebral cortex compared to NC. To be specific, the left OFC showed decreased FC with the right superior temporal gyrus (STG), bilateral postcentral gyrus, bilateral DLPFC, left fusiform gyrus, right occipital superior lobe, bilateral dACC, bilateral supplementary motor area (SMA), left lingual gyrus, right amygdala, right precuneus and bilateral dorsal striatum in IGD participants compared with NC. Participants with IGD also showed reduced FC from the right OFC to left lingual gyrus, right inferior temporal gyrus, right fusiform gyrus, bilateral STG, left postcentral gyrus, left precentral gyrus, right amygdala, left dorsal striatum, right dACC, right SMA, right posterior cingulate cortex (PCC) and right DLPFC than did NC.

**Table 2:** Brain regions showing decreased activation from seed regions in internet gaming disorder (IGD) participants compared to normal controls (NC).

seed	Regions	Peak MNI (mm)			T-value	voxels
		x	y	z		
**L. OFC**						
	R. Superior temporal gyrus	54	−26	0	5.42	1364
	B. Postcentral gyrus	68	−10	14	5.36	617
		−54	−20	18	5.18	189
	B. DLPFC	34	50	22	5.26	309
		−38	46	22	4.17	530
	L. Fusiform gyrus	−46	−42	−16	5.36	717
	R. Occipital superior lobe	24	−74	32	5.02	480
	B. dACC	2	0	44	4.88	988
		−12	8	42	3.61	807
	R. SMA	4	0	64	4.65	1099
	L. Lingual gyrus	−14	−62	−12	4.33	656
	R. Amygdala	18	2	−22	4.00	295
	R. Precuneus	6	−56	70	3.44	214
	B. Dorsal striatum	−24	−8	12	3.62	149
		28	−10	12	3.64	124
**R. OFC**						
	L. Lingual gyrus	−16	−62	−12	5.05	500
	R. Inferior temporal gyrus	64	−26	−26	4.99	178
	R. Fusiform gyrus	32	−64	−20	4.86	689
	B. Superior temporal gyrus	−62	−6	6	4.89	402
		66	2	4	4.37	566
	L. Postcentral gyrus	−26	−42	74	4.80	685
	L. Precentral gyrus	−50	2	52	4.75	194
	R. Amygdala	22	4	−20	4.62	295
	L. Dorsal striatum	−26	−8	10	4.18	353
	R. dACC	4	0	44	3.69	232
	R. SMA	2	−10	64	3.99	363
	R. PCC	16	−38	44	3.86	123
	R. DLPFC	34	50	20	3.77	124

Clusters with peak-level and FDR-corrected p < 0.05 with more than 100 voxels are reported.

Abbreviations: MNI, Montreal Neurological Institute coordinates; L., Left; R., Right; B., Bilateral; OFC, Orbitofrontal cortex (BA11); dACC, Dorsal anterior cingulate cortex; SMA, Supplementary motor area; DLPFC, Dorsolateral prefrontal cortex; PCC, Posterior cingulate cortex; FDR, False discovery rate.

#### Correlation between Functional connectivity and impulsivity

**Figure 2:**
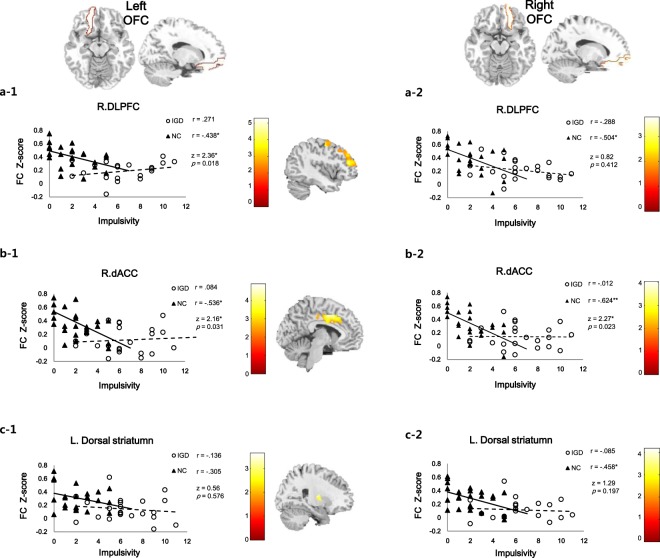
Correlation between the functional connectivity strength from the orbitofrontal cortex (OFC) to each regions of interest (ROIs) and dysfunctional impulsivity. There was a negative correlation with impulsivity and FC from the bilateral OFC to each ROIs such as right DLPFC (**a-1,a-2**), right dACC (**b-1,b-2**) and left dorsal striatum (**c-1,c-2**) in the NC (*p < 0.05, **p < 0.001).

As drawn in Fig. 2, the relationship between FC z-score and impulsivity score was significantly correlated with FC from the left OFC to right DLPFC, from the left OFC to right dACC, from the right OFC to right DLPFC, from the right OFC to right dACC, and from the right OFC to left dorsal striatum in the NC (Fig. 2: a-1, r = −0.438, *p* = 0.039; b-1, r = −0.536, *p* = 0.021; a-2, r = −0.504, *p* = 0.024; b-2, r = −0.624, *p* = 0.007; c-2, r = −0.458, *p* = 0.036), but not in IGD participants (a-1, r = 0.271, *p* = 0.667; b-1, r = 0.084, *p* = 0.850; a-1, r = −0.288, *p* = 0.667; b-2, r = −0.012, *p* = 0.956; c-2, r = −0.085, *p* = 0.850), respectively. The relationship between FC from the left OFC to left dorsal striatum and impulsivity score did not show any correlation in the NC (r = −0.136, *p* = 0.850) and also in IGD participants (r = −0.305, *p* = 0.147).

## Discussion

In this study, we examined the alteration of the cortico-striatal networks of internet gaming users using resting-state fMRI. By selecting the OFC as a seed region, we investigated changes in the brain connectivity with the other regions of the brain in IGD compared with the NC group. Furthermore, the relationship between the OFC connectivity and impulsivity level was examined. This study demonstrated that the IGD participants exhibited significantly lower FC from the bilateral OFC to overall brain regions compared to NC, with no significant differences in demographic characteristics across groups. In addition, it was confirmed that the FC strength of frontostriatal regions was linked to impulsivity in the NC, not in IGD participants. The correlation analysis has revealed that the increased frontostriatal connectivity, the OFC with dACC, the OFC with DLPFC and the OFC with dorsal striatum, was related to the impulse control in the NC.

In terms of the connectivity patterns of IGD participants, seed-based group-level analysis illustrated reduced connectivity between the OFC and cerebral cortex at large when compared to the NC. In previous studies, the altered brain connectivity in IGD over several brain regions including frontal regions were reported, which implies that a specific IGD characteristic is associated with the altered frontal FC^22,51–53^. Substance and behavioral addiction studies have mentioned for the role of the OFC in executive function such as decision-making and behavioral control among prefrontal cortex^54–56^. The OFC is engaged in decision-making by evaluating the reinforcers’ values and determining the action based on the predicted outcomes^57–59^. Likewise, human imaging studies figured out that the OFC is in charge of behavior control by selecting appropriate behavior following motivation for sustaining addictive substance or behavior^23,60^. In other words, dysfunctional OFC may contribute to risk-taking choice and this could be led to impulsive behavior^61,62^. Therefore, it is suggested that the decreased connectivity from the OFC to other brain regions would imply the impairment of cognitive control in IGD. Although the OFC takes an essential role in cognitive and impulse control as a prefrontal region, previous IGD studies regarding altered prefrontal network and its connection to impulsivity trait which is one of the important IGD characteristics^34,63^ had not included the OFC. As the OFC which accounts for impulse control in addiction was included in this study, it could be suggested that the results might provide the appropriate clue to modulate the maladaptive behavior from excessive use of internet game.

In particular, the GLM analysis results of this study exhibited decreased FC between the OFC and regions of interesting (ROIs): dACC, DLPFC, and dorsal striatum in IGD participants compared to the NC. It is known that frontal lobes, including the OFC, DLPFC, and dACC, involve in executive functions. The DLPFC, dACC, and dorsal striatum took their roles in maintaining attention, error detection and monitoring, and behavior regulation, respectively^64–66^. Previous studies have determined that the OFC and DLPFC were connected to goal-directed decision-making and self-control integrating associative information^60,67^. The OFC and ACC are commonly come up together in addiction studies for involving in higher-order cognitive along with craving control and response inhibition^68^. The interconnection between the OFC and dorsal striatum was deeply associated with compulsive behavior regulation in addiction connected with dopaminergic system^53,69,70^. Therefore, it could suggest that the weakened FC from the OFC to DLPFC, dACC, and dorsal striatum is linked to impairment in decision-making and self-control in IGD compared to NC. In addition, the less connectivity between the OFC and other regions could be associated with dysfunction in cognitive control and could be vulnerable to the impulsive use of internet game.

This study showed that the decreased FC in the frontostriatal network was associated with impulsivity control in the NC, however, IGD participants did not show relationship between frontostriatal connectivity and impulsivity control. In previous studies, it was reported that neural mechanisms of impulsivity was involved the PFC, the OFC and striatum, which are integrated into frontostriatal network^15,71^. Indeed, the NC has played internet game regularly without addictive symptoms. Therefore, it might be supposed that these healthy gamer could control their game pattern and keep their impulsivity control level. On the contrary, IGD participants showed less FC in frontostriatal compared to the NC, and it seemed that the weakened frontostriatal connectivity could not influence upon impulsivity control. Considering the role of frontostriatal network in impulse control, excessive internet game use might be induced regulatory failure of frontostriatal connectivity linked to impulsivity regardless with individual impulsivity score. Therefore, this study suggests that maladaptive behavior from excessive use of internet game might be induced regulatory failure of frontostriatal regions. In addition, this finding concerning brain circuitry of impulsivity could provide direction for behavioral treatment approaches of impulse control in IGD.

## Limitations

The study has at least three limitations. (1) The result is not accounted for the direct relationship between FC and impulsiveness since the cognitive function regarding impulse control was examined only with self-rating scale. Hence, it could be important to assess FC connected to impulsivity by performing behavioral tasks for detailed study. (2) Various factors that could mediate variables may not be fully reflected although we examined participants’ internet game usage pattern such as a played game genre, spent hours or costed money for internet gaming, and their comorbidity. Regarding that not everything could be considered as factors, further work is needed to examine the interplay among internet game usage, a psychological trait of IGD, and brain connectivity. (3) Lastly, this cross-sectional result cannot account for causation. Further studies using longitudinal data could verify the direct relationship and if the altered FC is a transitional phenomenon or a permanent state.

## Methods and Materials

### Participants

Participants were recruited by an online survey company. They were asked about their game usage routine and if they were interested in Magnetic Resonance Imaging (MRI) research. Based on the answers from the online survey, secondary screening was conducted to verify if they were qualified for the research. Participants answered on the self-reporting survey about their usage of internet games, including usage hours, amount of money spent on internet games, and the age at which they started the online game. Background information, including age, gender, level of education and their habitual behaviors for alcohol, nicotine and drug use were also queried on the survey. Participants who are currently taking psychiatric drugs was excluded.

Forty-six participants were included in this research, excluding adolescents, women, to minimize menstrual cycle effects on the neuroimage^72,73^ and because the prevalence of IGD is greater in men than women, and distorted image samples among the respondents to the survey. We had informed participants to not use internet game, alcohol and caffeine prior to 24 hours before MRI assessment. The sample comprised 22 males who were considered as IGD (28.27 ± 5.33 years) and 24 age-matched male controls (28.17 ± 5.93 years) who had played internet games at least once within a year. This research was approved by the Institutional Review Board at the Seoul St. Mary’s Hospital, Seoul, South Korea. Each participant was told the main purpose of the study and gave written informed consent after fully understanding the purpose of the research. The study was conducted in accordance with approved guidelines and regulations.

### Assessments

#### IGD-scale

To assess the problematic use of internet game, we used the self-reported Internet Gaming Disorder scale, which is based on the *Diagnostic and Statistical Manual for Mental Disorders* (DSM-5). This scale showed reliability and good criterion-related validity^74^.

#### Mini-International Neuropsychiatric Interview (MINI)

Participants were interviewed by a clinician to verify if they currently have psychiatric comorbidity such as neurological illness, schizophrenia, bipolar disorder or major depression^75^.

#### Wecchsler Adult Intelligence Scale (WAIS)

The Korean version of the WAIS was administered to assess the intelligence quotient (IQ) of all participants and to verify whether intelligence varied with the internet game usage status^76^.

#### Dickman Impulsivity Inventory (DII)

Dickman proposed two types of impulsivity: functional and dysfunctional impulsivity. Functional impulsivity presents a positive view of passion and risk-taking tendency with little forethought in optimal situations, while dysfunctional impulsivity presents a negative view of not having plans, not maintaining attention, and an absence of the goal, with less forethought in difficult situations^77^. We used a dysfunctional impulsivity inventory with 12 items adopted from DII to measure the dysfunctional impulsive behavioral approach of excessive internet game user. This self-reported questionnaire reported Cronbach’s alphas of 0.69.

### Image acquisition

Functional and structural MRI data were acquired using a 3T MAGNETOM Verio MRI system (Siemens, Erlangen, Germany) equipped with an 8-channel head coil. Participants’ heads were cushioned with attached earmuffs. Participants were instructed to stare at a cross fixation during resting-state fMRI to prevent over-movement of their eyes. Two hundred frames (volumes) of resting-state functional images were obtained using a T2*-weighted gradient echo-planar imaging sequence (repetition time [TR] = 2000 ms, echo time [TE] = 30 ms, 28 slices, slice thickness = 4 mm, no gaps between slices, flip angle = 90°, voxel size = 2 × 2 × 4 mm, image matrix = 124 × 124, field of view [FOV] = 192 mm). Structural images were acquired using a three-dimensional T1-weighted gradient echo sequence (TR = 2300 ms, TE = 2.52 ms, slice thickness = 1 mm, flip angle = 9°, voxel size = 1 × 1 × 1 mm, image matrix = 224 × 224, FOV = 256 mm^2^).

### Data analysis

For image preprocessing and statistical analysis, we used Statistical Parametric Mapping software 8 (SPM8, Wellcome Department of Imaging Neuroscience, London, UK) running on MATLAB R2015a (Mathworks, Sherborn, MA, USA) and Data Processing Assistant for Resting-State fMRI (DPARSF) software^78^. All statistical analyses were carried out with the Statistical Package for Social Sciences version 21.0 for Windows.

#### Preprocessing

Before aligning a series of images, the first five images of resting-state functional images were excluded to eliminate magnetic saturation effects. A total of 195 resting-state functional images were aligned for each participant to correct head motion errors. The T1-weighted structure image was segmented into white matter, gray matter and cerebrospinal fluid using the Montreal Neurological Institute (MNI) space skull-strip image template. The realigned functional images were co-registered on the T1-weighted image of the same participant. The motion-corrected functional volumes were normalized to the MNI space and resampled to 3 × 3 × 3 mm^3^ voxels. Functional data images were smoothed with a Gaussian Kernel of 6-mm full-width at half-maximum, bandpass filtered (0.009–0.08 Hz), and linearly detrended. Signals from rigid body 6 motions, white matter, cerebral space fluid and global motion were removed.

### Statistical analyses

We set each of the bilateral OFC as the seed regions and correlated the reference signal from the seed region with the signal from every voxel within the brain to acquire a FC map. Automated anatomical labeling (AAL) map^79^ was used to define the seed and ROIs. We used a two-sample t-test of the FC map derived from each seed to compare the group difference. The results were considered statistically significant if they had False Discovery Rate *p* values below 0.05 with an extent threshold of 100 voxels. Among the regions showing altered FC between groups, we selected the right dACC, right DLPFC and left dorsal striatum as ROIs where were shown the same in bilateral seed regions. The regions were selected because the neural changes of the regions were associated with impulse control^23,35,43,60,80^. Correlation analysis was conducted for impulsivity and the FC z-values between seed and ROIs: from the bilateral OFC to the DLPFC, dACC and dorsal striatum. FDR Multiple comparison corrections were conducted to the value of correlation between FC z-value and impulsivity score using Benjamini-Hochberg procedure. The correlations were then analyzed with Fisher’s r-to-z transformation to convert the correlation coefficients to z-scores for seeing the correlation proneness between FC strength and impulsivity.
